# Nasal endoscopic evaluation of children and adolescents with cystic fibrosis

**DOI:** 10.1016/S1808-8694(15)30541-3

**Published:** 2015-10-19

**Authors:** Letícia Paiva Franco, Paulo Augusto Moreira Camargos, Helena Maria Gonçalves Becker, Roberto Eustáquio Santos Guimarães

**Affiliations:** 1 Master's degree in pediatrics, Medical School of the Federal University of Minas Gerais -UFMG. Otorhinolaryngologist at the UFMG hospital; 2 Post-doctorate degree, Service de Pédiatrie et Pneumologie Pédiatrique do Hôpital d'Enfants Armand-Trousseau/Faculté de Médecine Saint-Antoine/Université Pierre et Marie Curie, PARIS VI. Full professor in the Pediatrics Department, Medical School of the UFMG. Coordinator of the Doctorate in Medicine Unit and the Cystic Fibrosis Multidisciplinary Team at the UFMG Teaching Hospital; 3 Doctoral student in medicine-ophthalmology, Medical School of the UFMG. Adjunct professor at the Medical School of the UFMG; 4 Associate Professor in the Ribeirão Preto Medical School of the Federal University of São Paulo. Adjunct professor at the Medical School of the Federal University of Minas Gerais

**Keywords:** endoscopy, cystic fibrosis, polyposis

## Abstract

The main otorhinolaryngological manifestations of CF are chronic rhinosinusitis and nasal polyposis, with different clinical presentations.

**Aim:**

To characterize children and adolescents with cystic fibrosis through a questionnaire, an ENT clinical examination and nasal endoscopy.

**Study design:**

Cross-sectional clinical descriptive.

**Material and Method:**

Assessment of 100 children and adolescents with cystic fibrosis through a specific questionnaire, ENT physical examination, nasal endoscopy and endoscopic staging of nasal polyps.

**Results:**

The most frequent symptoms were: cough (45%), oral breathing (44%), sleep disorders (42%) and nasal obstruction (37%). Twenty-eight patients (28%) had purulent nasal discharge, and 41% had medial bulging of the nasal lateral wall. Nasal polyps were identified in only 14% of cases, none were obstructing.

**Conclusion:**

The questionnaire, clinical examination and especially nasal endoscopy lead to a detailed assessment of the nasal characteristics of children and adolescents with cystic fibrosis. Some findings were discordant with the literature, particularly the low prevalence of nasal polyps, and appear to be related to specific characteristics of the population studied. The best characterization of this group of patients, from the ENT standpoint, contributes to an appropriate multidisciplinary approach.

## INTRODUCTION

Cystic fibrosis (CF) is the most common inherited autosomal recessive disorder in Caucasians.1-6 Its prevalence ranges from 1:6.000 to 1:2.000 of live births in white populations of developed countries;^1^, ^3^, ^4^, 6, 7, 8, 9 it is rare in black (1:30.000) and Asian (1:90.000)^10^, ^11^ populations. In Brazil, the incidence ranges from 1:9.500 in Parana state,^12^ to 1:8.700 in Santa Catarina state,^13^ and 1:10.000 in Minas Gerais state.^14^

This disease results from mutations in the cystic fibrosis transmembrane conductance regulator gene (CFTR), located on the q31 region of the long arm of chromosome 7.^15^, ^16^, ^17^ This gene, which was described in 1989,^15^, ^16^, ^17^ encodes for a protein that functions as a chloride channel; dysfunction of this channel results in altered sodium, chloride and water transport across the apical membrane of epithelial cells in the respiratory tract and in exocrine glands. Altered salt and water flow result in loss of hydration of exocrine gland fluids and altered viscoelasticity of mucins.^5^, ^6^, ^18^, ^19^

Most patients with CF (over 90%)^3^, ^6^, ^18^, 20, 21, 22, 23, 24, 25 develop chronic and recurring rhinosinusitis, with or without nasal polyps. Altered mucus composition and viscoelasticity results in decreased mucociliary clearance and obstruction of paranasal sinus drainage ostia, facilitating local inflammation, hypoxia and increased carbon dioxide partial pressure. Mucosal edema ensues, compromising ciliary function and favoring bacterial colonization, usually by Staphylococcus aureus and Pseudomonas aeruginosa.^3^, ^6^, ^18^, ^26^

Nasal polyposis in CF patients were first described in 1959;^27^ to date, little is known about its pathophysiology.^28^, ^29^, ^30^ The frequency of nasal polyposis varies in different populations, and depends on the evaluation technique.^26^, ^29^,^31^, ^32^, ^33^, ^34^, ^35^, ^36^, ^37^, ^38^, ^39^, ^40^, ^41^, ^42^

It is thought that nasosinusal involvement may worsen pulmonary manifestations;^20^ thus, otorhinolaryngologists have become more involved in evaluating these patients. Recent studies in Brazil^26^, ^40^, ^41^ have shown a growing concern with characterizing in more detail the nasosinusal findings of CF patients, because this disorder is genetically very heterogeneous, with many types of mutations and a wide variety of clinical presentations,^28^ which may be explained by specific phenotypic features of the Brazilian population and even within populations of any given region in this country.

The aim of this study was to characterize the nasosinusal findings in child and adolescent patients with CF in the state of Minas Gerais, using a questionnaire (clinical history), the physical examination, and nasal endoscopy nasal.

## MATERIALS AND METHODS

The Institutional Review Board of our institution evaluated and accepted the research design and the free informed consent form (protocol number: 177/2002). All patients and/or their caretakers agreed to participate and signed the free informed consent form.

We conducted a cross-sectional descriptive clinical study consisting of a guided clinical history, a full otorhinolaryngological physical examination, and nasal endoscopy of 100 patients aged from 7 months to 18 years, with a diagnosis of CF and varying degrees of respiratory involvement. All participants had clinical findings compatible with and a diagnosis of CF, confirmed by the sweat test (Gibson and Cooke method^43^) according to the guidelines of the Cystic Fibrosis Foundation.^8^

Patients were seen in the Pediatric Pneumology and Otorhinolaryngology Units of the hospital from July 2002 to January 2004. Hospitalized patients with a diagnosis of active upper airway infection, using systemic antibiotics or topical nasal corticosteroids, were assessed three to six months later, when these exclusion criteria were no longer present.

Nasal endoscopy was done with a flexible 3.2 mm diameter endoscope (Machida Endoscopy Co., Japan) after topical nasal anesthesia with neutocaine; in some cases, rigid endoscopy was done with a 4 mm diameter 00 angle endoscope (Fiegert Endotech, Germany).

Nasal polyps were staged according to Lund and Kennedy's^44^ endoscopic staging criteria, which evaluated the following parameters: nasal mucosa edema, presence of discharge, and presence of polyps. Each of these parameters were assessed bilaterally and scored from 0 to 2, as shown on Frame 1. The total score (0 – 12) is the sum of scores of each side.

**Frame 1:** Endoscopy scores in the Lund-Kennedy method.

Features	Right nasal cavity	Left nasal cavity
Polyp (0, 1, 2)		
Edema (0, 1, 2)		
Discharge (0, 1, 2)		
Total		

Notes:

Polyps: 0 - absent, 1 - restricted to MM, 2 - extending to the nasal cavity

Edema of mucosa: 0 - absent, 1 - mild/moderate edema, 2 - polypoid degeneration

Discharge: 0 - absent, 1 - hyaline, 2 - thickened and/or mucopurulent

Statistics consisted of a descriptive analysis, confidence intervals (calculated using the EpiInfo 2004 software), a comparison of means by the Kruskal-Wallis test, and parametric tests (“t” test and analysis of variance or ANOVA). Means were considered as statistically significant when the p value (“t” test, ANOVA, and the Kruskal-Wallis test) was below 0.05 (or 5%).

## RESULTS

The study sample comprised 61% of male patients with ages ranging from seven months to 18 years (mean: 8.4 years); 50% were aged below eight years, and 75% were aged below 11 years. Most of the patients (59%) were brown-colored; there were no black or Asian patients.

The most frequently reported (by patients and/or caretakers) upper airway complaints were coughing, predominant mouth breathing, sleep disorders (characterized as agitated sleep, snoring, and repeated awakenings), nasal block, halitosis, headaches, and rhinorrhea, as shown on Table 1. None of these symptoms were present in 21% of patients at the time of the evaluation.

**Table 1:** Distribution of symptoms in the sample of patients.

Variables	%	IC 95%
Coughing	45,0	34,5 – 54,8
Mouth breathing	44,0	33,5 – 53,8
Sleep disorders	42,0	31,6 – 51,8
Nasal obstruction	37,0	26,9 – 46,6
Halitosis	33,0	23,3 – 42,5
Headache	30,0	20,6 – 39,3
Rinorrhea	29,0	20,6 – 39,5
Dysphonia	20,0	12,8 – 29,5
Intolerance for physical activity	16,0	9,5 – 24,9
Daytime drowsiness	16,0	8,7 – 23,8
Periorbitary pain	11,0	5,0 – 17,8
Facial pain	10,0	4,2 – 16,6
Anosmia	2,0	0,2 – 7,1

The physical examination and endoscopy revealed that 36% of patients had a normal middle meatus (MM) bilaterally. The sphenoethmoidal recess (REE) could not be evaluated in most patients (51% to the right and 58% to the left) since this structure is undeveloped in children and hard to evaluate in the presence of septal deviations, polyps, or medial bulging of the lateral wall of the nose. Thirty patients had bilaterally normal REEs. Medial bulging of the lateral wall of the nose was found in 41 patients (bilateral in 36 of these patients). Patients in which the auditory tube ostia were not seen had adenoid hyperplasia, which caused obstruction (Table 2).

**Table 2:** Distribution of nasal endoscopic findings.

Variables	Nasal cavity (%)		
	Right	Left	Bilateral
Nasal mucosa			
Congestion	57,0	54,0	24,0
Hyperemia	10,0	10,0	9,0
Mucoid discharge			
Floor of nose	44,0	48,0	20,0
Middle meatus	33,0	28,0	15,0
Sphenoethmoidal recess	1,0	1,0	0,0
Mucopurulent discharge			
Floor of nose	12,0	14,0	10,0
Middle meatus	17,0	21,0	13,0
Sphenoethmoidal recess	3,0	3,0	1,0
Other findings			
Nasal polyps	10,0	9,0	5,0
Medial bulging of lateral wall	41,0	36,0	36,0
Free auditory tube ostium	86,0	83,0	79,0
Nasal septal deviation	…	…	19,0

There was no statistically significant association (p = 0.68) between age and the presence of medial bulging of the lateral wall of the nose.

Nasal endoscopy revealed mucoid discharges in 58% of patients, particularly on the floor of nose; mucopurulent discharges were found in 28% of patients (25% in the MM). There was an association between the presence of nasal discharges on endoscopy and reports of rhinorrhea (p = 0.05), as expected. The presence of nasal discharge was more common in patients aged from 6 to 18 years compared to patients aged 5 years or less.

Endoscopy revealed nasal polyps in fourteen patients (14%), of which five had bilateral polyps (35.7%). There was no statistically significant difference in the side of the nose where polyps were found (p = 1.0) or in the presence of mucopurulent discharges (p = 0.67). Table 3 shows the statistically significant association between nasal polyps and mucopurulent discharges (p = 0.02) and between nasal polyps and discharges of any kind (p = 0.02).

**Table 3:** Comparison of the presence of polyps, symptoms, sex and age.

Nasal polyps							
	Yes		No				
	N	%	N	%	Valor-p	OR	IC 95%
Mucopurulent discharge							
Yes	8	28,6	20	71,4	0,02*	4,40	1,18-16,77
No	6	8,3	66	91,7		1,0	
Mucoid discharge							
Yes	12	17,9	55	82,1	0,13*	3,38	0,64-23,78
No	2	6,1	31	93,9		1,0	
Any discharge							
Yes	14	18,7	61	81,3	0,02*	…	
No	0	0,0	25	100,0		1,0	
Sex							
Male	5	8,2	56	91,8	0,07	0,3	0,08-1,10
Female	9	23,1	30	76,9			
Age							
< 6 years	6	22,7	21	77,8		1,0	
6 – 11 years	6	12,2	43	87,8	0,33*	0,49	0,12-1,99
? 12 years	2	8,3	22	91,7	0,25*	0,32	0,04-2,07

There were no associations between sex or age and nasal polyps (p = 0.07), as seen on Table 3. However, 50% of nasal polyp cases were diagnosed in children aged 6 years or less, and no polyps were found in patients aged 16 years or above. Of 14 patients with polyps, 9 (64.3%) were aged between 4 and 12 years.

**Chart 1:**
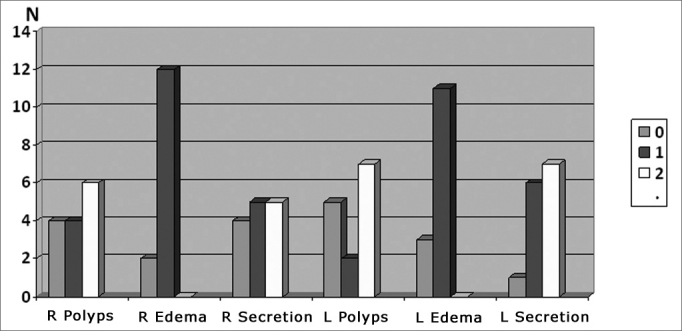
Endoscopic staging (Lund-Kennedy) of 14 patients with nasal polyps.

No patient presented obstructing polyps. The Lund-Kennedy staging^44^ of 14 patients with nasal polyps is shown on Chart 1 (mucosal edema, nasal discharge, and presence and size of polyps, bilaterally.

## DISCUSSION

Most of the papers cited below are by European and North American authors; some have studied both children and adults with CF. They thus reflect a different population to that of our study. There are few published studies on the features of CF populations in developing countries, many of which have smaller samples compared to our study.

### Clinical findings

The most frequently reported symptoms in children and adolescents with CF reported in our study were coughing, the most frequent symptom (45%), mouth breathing (44%), agitated sleep (42%), nasal block (37%), halitosis (33%), headaches (30%), and rhinorrhea (29%). Mouth breathing and sleep disorders are closely related with nasal block.^15^ Two patients only reported anosmia, and about 10% reported periorbitary or facial pain. The reported frequency of these symptoms in CF patients is very variable in the literature, although generally higher than the frequency in our study. Recent European and North American studies have shown that the symptoms most frequently associated with CF are coughing (in about 60%),^29^ nasal block (in 60 to 90.5%),^3^, ^29^, ^45^,^46^ rhinorrhea (in 20 to 85.7%),^3^, ^29^, ^36^, ^45^, ^46^ headaches (in 22.6 to 71.4%),^3^, ^29^, ^46^ facial pain (in 15 to 57.1%),45,46 and sleep disorders with agitation, snoring and daytime drowsiness (in 37%), especially in patients with severe nasal block.15 In Brazil, Boari and Castro Jr.^42^ found that except for coughing, symptoms of rhinosinusitis occurred infrequently. These variations probably reflect the small samples and different age groups in these studies.

An important point is that although some studies have shown that up to 100% of CF patients had pansinusopathy evidenced on facial sinus computed tomography,^3^, ^6^, ^18^, 20, 21, 22, 23, 24, 25 20% of these patients complained of no symptoms, and 36% had no alterations in the middle meati bilaterally. Other studies have also shown a lack of correlation between tomographic findings and clinical manifestations. Moss and King^47^ found that a relatively low rate of nasal complaints occurs because patients or caretakers pay more attention to the severe findings of the disease; patients appear to “adapt” to chronic nasal symptoms and lack knowledge about or underestimate how much nasosinusal manifestations may affect the progression of respiratory conditions.

### Nasal endoscopy

There is a wide variety of endoscopic findings in patients with CF, and a tendency for more diagnoses of nasal polyps as we move forward in time. From 1961 to 2005, the prevalence of nasal polyps increased from 5 to 57%.^26^, ^29^, 31, 32, 33, 34, 35, 36, 37, 38, 40, 41, 42 Explanations include increased survival rates of CF patients in developed countries, the selection of patients, sample sizes, different age groups in these studies, and especially the more recent routine use of nasal endoscopy as a diagnostic tool.^3^, ^19^, ^48^ Brihaye et al.^29^ showed that over 25% of polyps were not diagnosed in the clinical examination with anterior rhinoscopy without nasal endoscopy in CF patients. Only nasal endoscopy can provide an adequate view of the middle meatus and the posterior half of the nose.^4^, ^21^, ^49^ Boari and Castro Jr^42^ compared clinical, tomographic and endoscopic findings and found that nasal endoscopy helped significantly in evaluating chronic rhinosinusitis and nasal polyps in CF patients; endoscopy accurately characterized the nasosinusal status.

Nasal polyps were found in only 14% of children and adolescents with CF in our study, although our assessment method was similar to that in recently published papers. Studies of children and adolescents only have shown varying frequencies of nasal polyps: 22.2% of 27 CF patients by Denoyelle et al.^36^ in France, 57% of 23 CF patients by Yung et al.^19^ in England, and 32.6% of 89 patients by Cimmino et al.^6^ in Italy. Schmitt et al.^41^ retrospectively evaluated the medical files of 893 children with CF - the largest sample of this disorder in the literature - and found nasal polyps in only 5% of subjects.

Brazilian studies have also shown a significant variation in the diagnostic frequency of nasal polyps in CF patients. Weber and Ferrari^40^ found nasal polyps in 39.1% of CF patients in a sample of 23 patients aged from one year and nine months to 22 years and 8 months. Boari and Castro Jr.^42^ used nasal endoscopy and found polyps in only three (8.82%) of 34 CF patients aged from six to 22 years. Sakano et al.^26^ used rigid endoscopy under general anesthesia and found nasal polyps in 36% of 50 patients aged over 2 years; this method may have increased the diagnostic accuracy for polyps, including very small ones. These three studies evaluated children within the same geographical area (state of Sao Paulo).

Given the ample genetic heterogeneity of CF, with many mutations and a variety of clinical presentations,^28^ a low prevalence of nasal polyposis in our study compared to studies in developed countries may be explained by the specific genotypic features of each sample population. One may also suspect that CF is underdiagnosed in Brazil, and that early death of patients with respiratory complications - possibly with nasal polyps - may occur, given that in this country the mean survival age of CF patients is lower than that in developed countries, where it may reach 31.6 years.^6^, ^50^ A recent Brazilian study by Alvarez et al.^51^ revealed that the mean survival of 104 CF patients in that study was 18 years and four months after the diagnosis.

Our results shown that all patients with nasal polyps also had nasal discharges, although there was no relation between the presence of nasal polyps and symptoms such as coughing, rhinorrhea, mouth breathing, agitated sleep, headaches, and nasal block, even though certain authors^1^, ^3^, ^24^, ^28^ have described nasal block as a typical symptom in CF patients with nasal polyps. Gentile and Isaacson^28^ compared 19 CF patients aged from six to 25 years, with and with no nasal polyps, and found that the most frequent complaint in patients with polyposis was nasal block. Henriksson et al.^5^ also found no differences among patients with and with no nasal polyps concerning symptoms such as rhinorrhea, nasal block, and mouth breathing. Kennedy and Loury^52^ had found no parallel between nasal symptoms and the occurrence of nasal polyps.

In the present study we found no statistically significant association between nasal polyps and age; however, 64.3% of patients with polyps were aged from 4 to 12 years, concurring with Jaffe et al.'s^53^ and Sakano et al.'s^26^ reports (88.89% of cases of nasal polyps in patients below age 15 years). On the other hand, Brihaye et al.^3^ reported that the prevalence of polyps in younger children was significant, since (50%) of 14 children and adolescents with nasal polyps were aged six years or less; the youngest child with a nasal polyp was aged eight months, with the differential diagnosis being made by computed tomography of the nose and facial sinuses. No other author has described nasal polyps in CF patients aged below one year and four months.

It is thought that medial bulging of the lateral wall of the nose results from intraluminal pressure exerted by polyps and/or viscous secretions retained in the paranasal sinuses, especially in younger children in which the lateral wall is more elastic than in adults.^3^, ^21^ Medial bulging of the lateral wall of the nose was found in 41% of our sample, which concurs with other published results (12 to 76%).^3^, ^29^, ^46^ However, there was no statistically significant relation between medial bulging of the lateral wall of the nose and the age of patients in this study.

### Endoscopic staging of nasosinusal polyps

Endoscopic staging of nasal polyps is required to quantify disease severity and to assess the response to therapy. It is also possible to compare data with that of other authors, thus characterizing different groups of patients with nasal polyps. There is, however, consensus around a single and uniform staging method in the literature.^44^

Several authors, applying different staging methods, have reported variable prevalence rates of obliterating polyposis. A few studies reported the presence of many polyps in up to 70% of cases, while other studies found no CF patient with obstructing polyps.^3^, ^5^, ^6^, ^19^, ^29^

None of the 14 children with nasal polyps in our sample had obliterating polyps. Ten patients had nasal polyps to the right and 9 patients had polyps to the left. The maximum score, in the Lund-Kennedy^44^ staging method, was 10 and the minimum score was 2, which demonstrates the variable nature of nasal endoscopic findings in CF patients with nasal polyps.

Our findings show that the clinical and endoscopic nasal findings in 100 children and adolescents were in part different from those of CF patients in developed countries and in different regions of Brazil. There is a paucity of such information in Latin-American countries, and efforts should be made to increase our understanding of the specificities of our CF populations, to guide specific therapy and early public health measures based not only on international data. Future studies involving all major centers treating CF in Brazil with standard methods would be of great value in a multidisciplinary approach to these patients.

## CONCLUSION

This study led to the following conclusions in the sample population:

• the main otorhinolaryngological manifestations were coughing, mouth breathing, disordered sleep, nasal block, halitosis, headaches, and rhinorrhea; these findings were generally found at a lower proportion compared to other studies of patients with CF;
• nasal polyps were present in only 14% of patients; there were no cases of obstructing polyps; there was a significant association between the presence of nasal polyps and nasal discharge, but not between nasal polyps and age, or nasal polyps and symptoms such as nasal block, coughing and headaches;
• medial bulging of the lateral wall of the nose was seen endoscopically in a significant number of patients (41%);
• nasal endoscopy was carried out successfully in all 100 patients; it was performed with no difficulty and was deemed essential in the evaluation of the nose and sinus cavities of patients with CF, especially in patients with respiratory manifestations with no accurate diagnosis or difficult to control clinically, regardless of age.
